# Sustainable Bio-Based Polymer Adsorbents for Water Remediation: A Systematic Review of Functionalization Strategies, Performance Enhancement, and Circular Economy Integration

**DOI:** 10.3390/polym18192350

**Published:** 2026-09-26

**Authors:** Segundo Rojas-Flores, Moisés Gallozzo-Cardenas, Santiago M. Benites, Daniel Delfin-Narciso, Aníbal Alviz-Meza

**Affiliations:** 1 Facultad de Ingeniería y Arquitectura, Universidad Autónoma del Perú, Lima 15842, Peru; 2 Departamento de Ciencias, Universidad Tecnológica del Perú, Trujillo 13011, Peru; c21228@utp.edu.pe; 3 Escuela de Postgrado, Universidad Señor de Sipan, Chiclayo 14001, Peru; benitescasti@uss.edu.pe; 4 Grupo de Investigación en Ciencias Aplicadas y Nuevas Tecnologías, Universidad Privada del Norte, Trujillo 13011, Peru; daniel.delfin@upn.edu.pe; 5 Escuela de Posgrado, Universidad Continental, Lima 15046, Peru; aalviz@continental.edu.pe

**Keywords:** bio-based polymers, functionalized biopolymers, water remediation, pollutant adsorption, cellulose, chitosan, circular economy, systematic review, sustainable wastewater treatment, synthetic polymer integration

## Abstract

Global water scarcity affects over 57% of the world’s population, with industrial effluents discharging more than 200,000 tons of synthetic dyes and substantial heavy metal quantities annually. Conventional treatment methods remain energy-intensive and generate secondary toxic sludge, necessitating sustainable alternatives aligned with circular economy principles. This systematic review, conducted following PRISMA 2020 guidelines, analyzes 4626 studies from the Scopus database (2010–2026) using bibliometric analysis (RStudio/bibliometrix), network visualization (VOSviewer), and interactive graphics (Plotly). The field exhibits exponential growth (R_0_ = 96.5%, R^2^ = 91.1%), with publications concentrated in Materials Science (16%), Environmental Science (15%), and Chemistry (14%). Cellulose- and chitosan-based adsorbents demonstrate exceptional capacities: 1604 mg/g for mercury (thiolated chitosan) and 2100 mg/g for Congo red (cationic nanocellulose). A critical knowledge gap remains the limited integration of underutilized biomass with synthetic polymers such as PVDF—addressed in only 5 publications—despite hybrid systems achieving 92% circular readiness and 95% resource recovery. Combined experimental and computational approaches enhance adsorption performance by 20–50%, with materials retaining over 80% reusability after 10 cycles. Compared with conventional technologies, bio-based alternatives reduce energy consumption by 40% and CO_2_ emissions by 55% (0.043 kg CO_2_e/m^3^), while transforming agro-industrial wastes into high-performance adsorbents. These findings position functionalized bio-based polymers as technically, economically, and environmentally superior options for advanced water remediation, contributing to sustainable wastewater treatment and resource recovery within circular economy frameworks.

## 1. Introduction

The global water crisis remains one of the most critical challenges of the 21st century. By 2050, the world population is projected to reach between 9.4 and 10.2 billion, which will increase the demand for water systems by 22% to 34% [1]. Currently, over 57% of the global population experiences water scarcity for at least one month per year, and an estimated 5 million people die annually from consuming contaminated water [2]. This crisis is largely driven by the discharge of untreated industrial effluents containing highly persistent organic and inorganic pollutants. The chemical and textile industries are primary sources of environmental degradation [3]. Global synthetic dye production exceeds one million tons annually, of which approximately 20% is lost during processing and ultimately ends up in water bodies [4]. These dyes, particularly azo dyes, are highly stable and carcinogenic, even at low concentrations. At the same time, heavy metal pollution is equally alarming; smelting activities account for 40% of global emissions of metals such as Zn, Cd, and Pb, while the leather industry discharges nearly 40% of the chromium applied in its processes directly into wastewater [5,6]. In this context, functionalized bio-based polymers offer a viable pathway toward the circular economy. Although over 360 million tons of plastic are produced annually, bioplastics currently represent less than 1% of the total. The development of adsorbents derived from functionalized cellulose and chitosan has enabled removal efficiencies exceeding 90% for both metals and dyes, while also facilitating resource recovery and minimizing the generation of secondary toxic sludge [3,5].

The integration of biopolymers with nanotechnology for advanced water remediation has spurred significant innovation, achieving removal efficiencies of over 90% for metals and dyes [7]. Given the annual production of 1 million tons of dyes, with 20% ending up in effluents, chitosan- and cellulose-based adsorbents are proving essential [8]. These technologies enable the recovery of critical resources—such as lithium, with 99.9% efficiency—and reduce energy consumption by 40% and carbon emissions by 55% compared to conventional activated carbon systems [9]. For instance, Sánchez-Ferrer et al. (2026) used fir wood membranes modified with maleic anhydride to treat metal solutions; their results highlighted 99.9% removal of Li^+^ and 72% removal of Fe^3+^ with notable stability [10]. Similarly, Kashaudhan et al. (2026) developed a xanthate chitosan hydrogel to remove Co(II) and methylene blue from synthetic waters, achieving adsorption capacities of 386.7 mg/g and 150.58 mg/g, respectively, with high reusability [11]. Hanyny et al. (2026) employed a composite of red clay and wet olive pomace to remove methylene blue, reporting an adsorption capacity of 58 mg/g and photocatalytic degradation of 95% [12]. Dhivya J. et al. (2026) used a porous polymer template decorated with Cu_3_P–ZnWO_4_ nanocrystals to treat moxifloxacin, achieving a dissipation efficiency of ≥99.4% in just 20 min under visible light [13].

Conducting a bibliometric analysis and systematic review is essential for transparently mapping the scientific and technical evolution of bio-based polymers in water remediation [14]. The use of the Scopus database guarantees access to a comprehensive collection of high-quality literature, enabling the identification of citation networks and global collaborations [15]. The RStudio environment, a prominent platform for statistical processing, allows for the analysis of large datasets to identify emerging trends [16]. The use of packages such as bibliometrix and software such as VOSviewer is vital for visualizing co-occurrence maps that reveal the underlying knowledge structure in the field [17]. Incorporating Plotly adds an interactive layer to metric visualization, facilitating the dynamic communication of complex results [18]. This methodological approach enables the synthesis of findings on biopolymer functionality and supports the development of solutions aligned with the Sustainable Development Goals and the circular economy [19,20]. The knowledge gap centers on the scarcity of systems that effectively integrate renewable cellulose with synthetic polymers like PVDF—combining high removal capacity, mechanical stability, and a molecular-level understanding of the adsorption process. In addition, research on the use of underutilized biomass through integrated experimental and computational approaches remains limited.

The overall objective of this research is to conduct an in-depth review of the scientific literature on functionalized bio-based polymers applied to advanced water remediation, in order to map the evolution of knowledge, identify emerging trends, and evaluate the performance of these materials in pollutant adsorption. To this end, the study intends to address the following research questions: Q1: Analyze global scientific production on functionalized bio-based polymers for water remediation. Q2: Evaluate the state of the art of biopolymer-derived adsorbents (cellulose, chitosan, and others), with an emphasis on their removal efficiency for heavy metals and dyes, molecular interaction mechanisms, and advances in nanotechnology for advanced water treatment. Q3: Identify knowledge gaps and research opportunities in the integration of underutilized biomass with synthetic polymers (such as PVDF). Q4: Apply experimental and computational approaches to optimize the adsorption capacity, mechanical stability, and reusability of synthetic polymer materials. Q5: Synthesize findings on the environmental and energy impact of these technologies compared to conventional systems, highlighting their contribution to the circular economy through critical resource recovery, carbon emission reduction, and minimization of toxic sludge generation.

## 2. Materials and Methods

### 2.1. Data Source and Search Strategy

The systematic review and bibliometric analysis were conducted in accordance with the PRISMA 2020 guidelines to ensure methodological transparency, reproducibility, and rigor in the selection and synthesis of the scientific literature. *The review protocol was registered on the Open Science Framework (OSF) prior to data extraction to enhance transparency and reproducibility (available at: https://osf.io/8bcrm/overview?view_only=095f1d82b3d84d08b0e342a26898814f, accessed on 20 September 2026).* The Scopus database was chosen as the primary data source due to its comprehensive coverage of peer-reviewed literature across multiple scientific disciplines, including materials science, environmental chemistry, chemical engineering, and polymer science. Scopus provides extensive citation indexing and broad interdisciplinary coverage, making it particularly well-suited for mapping research fields that intersect several knowledge domains. Nonetheless, Scopus’s coverage is not exhaustive: literature published in languages other than English and research disseminated in regional or non-Scopus-indexed journals—particularly from Latin America, Africa, and parts of Asia—is likely underrepresented in this dataset. The bibliometric trends reported here should therefore be interpreted as characteristic of the internationally visible, predominantly English-language literature. Because this review relied exclusively on Scopus, we did not test whether the reported growth patterns or disciplinary distributions are sensitive to database choice; cross-database validation (e.g., against Web of Science or Dimensions) is recommended for future bibliometric studies in this field.

The search strategy was designed to capture the full scope of research on functionalized bio-based polymers for water remediation. The Boolean search string combined terms corresponding to five core conceptual dimensions: (1) functionalization (“functionalized” OR “modified” OR “tailored” OR “enhanced”); (2) bio-based materials (“bio-based” OR “biopolymer” OR “biodegradable” OR “renewable”); (3) polymer matrices (“polymer” OR “plastic” OR “material” OR “compound”); (4) aqueous systems (“water” OR “aqueous” OR “liquid” OR “hydro”); and (5) remediation processes (“remediation” OR “treatment” OR “purification” OR “decontamination”). This comprehensive query structure ensured the retrieval of relevant documents while maintaining adequate specificity. The search was performed on 1 July 2026, covering the period from 2010 to 2026, and yielded 4626 documents. It should be noted that the search strategy and inclusion criteria were designed to capture literature on heavy metal and dye removal, the two dominant pollutant classes in this field; consequently, adsorption of microplastics and pharmaceuticals/PPCPs—both emerging areas of growing relevance—was not systematically captured and appears only incidentally (e.g., ref. [21]). A dedicated systematic review of bio-based adsorbents for microplastic and PPCP removal is recommended as future work. A broader temporal search spanning 1974 to 2026 identified 5008 total records, providing historical context for the evolution of the field and enabling the analysis of long-term research trends.

### 2.2. Study Selection Process and PRISMA Workflow

The screening and selection process followed a systematic, multi-stage approach to minimize bias and ensure that only relevant studies were included. From the initial 5008 records identified, duplicate records (n = 10) were removed, records marked as ineligible by automation tools (n = 16) were excluded, and documents outside the target timeframe (n = 121) were eliminated prior to screening, leaving 4861 records for initial evaluation. During the screening phase, studies focused exclusively on synthetic polymers (e.g., PVDF, PES, PAN) without a bio-based component were excluded (n = 39), resulting in 4822 reports sought for retrieval. Of these, 8 reports could not be retrieved. Full-text eligibility assessment of the remaining 4814 reports led to the exclusion of studies on groundwater or soil remediation (n = 26), those focused exclusively on photocatalytic degradation without an adsorption component (n = 48), and gas-phase or non-aqueous adsorption studies (n = 114). The final review thus included 4626 studies, representing a robust dataset for comprehensive bibliometric and content analysis. This systematic workflow is illustrated in Figure 1 (PRISMA 2020 Flow Diagram), which documents each stage of the identification, screening, and inclusion process. The complete PRISMA 2020 checklist is provided in Supplementary Table S1, detailing all 27 items across the seven sections of the checklist framework.

### 2.3. Bibliometric and Statistical Analysis Tools

Quantitative data analysis was performed using the R programming environment (version 4.5.2) within RStudio (version 2026.01.0+392), an integrated development environment offering robust capabilities for statistical computing and data visualization. The bibliometrix package (version 5.4.1) was employed as the primary tool for comprehensive science mapping analysis. This package supports a complete bibliometric workflow, including data import from Scopus, descriptive statistics calculation, citation network construction, co-citation analysis, bibliographic coupling, and co-word analysis. Its functionality enabled the identification of publication trends, citation patterns, annual scientific production, the most influential authors and journals, and the evolution of key research themes over the 16-year study period. For network visualization and mapping of scientific landscapes, VOSviewer (version 1.6.21, released 12 June 2026) was used. This software is specifically designed for constructing and visualizing bibliometric networks based on co-occurrence data, including author collaboration networks, journal co-citation maps, and keyword co-occurrence analyses. VOSviewer’s ability to generate distance-based maps with intuitive visual representations facilitated the identification of research clusters, emerging topics, and the intellectual structure of the field. The software requires Java (version 8 or higher) for operation. To enhance the interactivity and dynamic communication of bibliometric metrics, Plotly (R package version 4.12.0) was integrated into the analytical workflow. This package enables the creation of interactive web graphics from ggplot2 objects and provides a custom interface to the plotly.js JavaScript library. The interactive visualizations allowed for dynamic exploration of publication trends, citation distributions, and thematic evolution patterns, facilitating a more nuanced interpretation of complex bibliometric data.

### 2.4. Analytical Framework and Data Interpretation

The methodological framework integrated multiple analytical approaches to provide a comprehensive understanding of the research field. Descriptive bibliometric indicators—including annual publication counts, citation metrics, and author productivity—were calculated to characterize the growth trajectory and impact of the field. Network analysis techniques, implemented through both bibliometrix and VOSviewer, were applied to construct co-authorship networks, keyword co-occurrence maps, and journal co-citation analyses. These methods enabled the identification of research clusters, collaborative patterns, and the conceptual structure of the knowledge domain. Thematic evolution analysis was conducted to track the emergence and transformation of research topics over time, revealing how the field has shifted from fundamental studies of biopolymer adsorption toward more applied research on nanocomposites, hybrid materials, and circular economy approaches. The integration of Plotly added a layer of interactivity to the visualization of metrics, facilitating the dynamic communication of complex results and supporting the identification of trends and gaps in the literature. This multi-tool analytical approach enabled the systematic synthesis of findings on biopolymer functionality, supporting the development of solutions aligned with Sustainable Development Goals and circular economy principles.

## 3. Result and Discussion

Given the substantial heterogeneity in experimental conditions across the 4626 included studies (adsorbent dose, initial pollutant concentration, pH, contact time, and functionalization method), a formal meta-analysis with pooled effect sizes, forest plots, and heterogeneity statistics (I^2^) was not performed. Instead, this section follows a narrative/bibliometric synthesis approach consistent with PRISMA 2020 guidance for reviews with high methodological heterogeneity: adsorption capacities are reported as the maximum values documented by the original authors under their optimal tested conditions, and should be interpreted as illustrative benchmarks of best-in-class performance rather than statistically pooled estimates. Figure 2 presents two complementary dimensions that characterize the current state and trajectory of the field of functionalized bio-based polymers applied to water treatment. Figure 2a illustrates the annual evolution of scientific output alongside the cumulative number of documents, fitted to an exponential model of the form y = y0 + A⋅exp(R0⋅x). The parameters obtained reveal exceptionally rapid growth; the growth coefficient R0 = 96.47 ± 0.99 and the high adjusted R-squared value (91.1%) indicate that the model explains more than 91% of the observed variability. This exponential behavior reflects that scientific interest in functionalized biopolymers has emerged over the past decade, rising from just a few annual publications before 2010 to over 600 documents in 2026. The steep slope observed from 2020 onward suggests that driving factors—such as the global water crisis, stricter environmental regulations, and advances in materials chemistry—have converged to position these adsorbents as a viable alternative to conventional activated carbons [21,22]. The cumulative number of documents exceeds 4600 over the analyzed period, providing a sufficient critical mass to undertake robust systematic reviews and bibliometric analyzes with statistical significance. Figure 2b displays the distribution of publications by subject area, revealing a marked interdisciplinary nature. Materials Science leads with 16% (1675 documents), closely followed by Environmental Science (15%) and Chemistry (14%). Chemical Engineering (12%) and Engineering (11%) complete the core cluster of disciplines, while Biochemistry (8%), Energy (6%), and Agricultural Sciences (5%) reflect the connection to biological processes and sustainability. The presence of Physics and Astronomy (4%) suggests that contributions are also coming from structural characterization and theoretical modeling. The remaining 9% encompasses areas such as medicine, pharmacy, and computer science, indicating emerging applications in contaminant detection and computational modeling. This distribution reveals that the field is not exclusive to polymer chemistry but is enriched by integrated approaches where materials synthesis, ecotoxicological assessment, process engineering, and life cycle assessment converge to offer circular solutions [23]. These findings support the interpretation that the exponential growth in publications has been primarily driven by the materials and environmental areas, which is consistent with the need to develop efficient and sustainable adsorbents [24]. However, the lower representation of areas such as Energy (6%) and Agriculture (5%) may point to research opportunities in the valorization of agro-industrial by-products and the integration of adsorption processes with bioenergy generation. Likewise, the high percentage of Chemistry and Chemical Engineering suggests that efforts are concentrated on surface functionalization, adsorption kinetics, and process scale-up, whereas the smaller contribution from Physics indicates that modeling molecular interactions and transport phenomena could be an area to strengthen [25]. Figure 2 confirms that the field has reached a level of maturity that justifies the present systematic review, while also highlighting emerging research niches—such as the integration of underutilized biomass and the development of hybrid materials with synthetic polymers (e.g., PVDF)—that align with the specific objectives set forth in this work [15].

Table 1 presents the most highly cited articles, characterized by their investigation of the sorption capacity of various celluloses, comparing the performance of natural materials against functionalized counterparts in the removal of heavy metals and industrial dyes. First, Bhatia [26] highlights that cellulose crystallinity is a critical factor in its recalcitrance, observing that the hydrolysis rate of amorphous cellulose is approximately 30 times higher than that of its crystalline fraction. Furthermore, the use of ionic liquids has demonstrated cellulose solubilities ranging from 5% to 20%, thereby facilitating its processing for novel applications. Suhas [27], for its part, highlights the low efficiency of natural cellulose, which exhibits Pb(II) removal capacities of only 10.78 mg/g, whereas functionalization with thiol groups raises this value to 28.67 mg/g, and mercerization enables values of up to 232 mg/g for the same metal. In the realm of cellulose-derived activated carbons, specific surface areas of 1317 m^2^/g and pore volumes of 0.608 cm^3^/g have been reported, optimizing the capture of organic contaminants. Regarding chitosan, Wang J. [28] documents that this modified biopolymer—specifically via CTS-g-PAA/GE hydrogels—achieves a maximum adsorption capacity (q_m_) of 781.25 mg/g for Pb(II). In the case of mercury, thiol-grafted chitosan reaches 1604 mg/g. Complementing this, Saheed [29] analyzes how crosslinking, although sometimes reducing porosity, improves mechanical stability; a graphene-chitosan composite exhibited a capacity of 384.62 mg/g for Congo Red, while Fe_3_O_4_-modified chitosan achieved 758 mg/g for methyl orange.

Advances in nanocomposites are detailed by Ghorai [30], who reported that a xanthan gum/nanosilica adsorbent (h-XG/SiO_2_) achieved removal efficiencies of 99.4% for methylene blue (Q_max_ = 497.5 mg/g) and 99.1% for methyl violet (Q_max_ = 378.8 mg/g). This process, driven by electrostatic interactions and hydrogen bonding, demonstrated efficient regeneration of 95.7% at pH 2. Regarding alginate, Wang B. [31] highlights its versatility when combined with other materials, achieving capacities of 2300 mg/g for methylene blue using graphene/alginate nanocomposites and 1727 mg/g for Pb(II) using sodium alginate-carboxymethyl cellulose beads. Nanocellulose represents a modern technological pillar. Voisin [32] explains that the formation of carboxylate groups via TEMPO oxidation or succinic anhydride treatment enables Pb(II) uptakes of 458 mg/g. Additionally, cationic nanocellulose grafted with hyperbranched polyethyleneimine has demonstrated extraordinary removal of 2100 mg/g for Congo Red. On the other hand, although oriented toward medicine, Viegas [33] provides a molecular design perspective by comparing polyoxazoline with PEG, noting that the former offers lower viscosity and higher loading capacity—properties that are transferable to the design of advanced adsorbents for pharmaceutical wastewater. The use of membranes and immobilized enzymes closes the treatment loop. Salehi [34] describes that chitosan/hydroxyapatite membranes can adsorb 296.7 mg/g of lead with equilibrium times of only 30 min. In the case of hexavalent chromium, epichlorohydrin-crosslinked chitosan reached a removal capacity of 1400 mg/g. In the biotechnological arena, Bilal [3] demonstrates that ligninolytic enzymes immobilized on sol–gel matrices or alginate-chitosan beads achieve decolorizations of up to 100% in just 5 h, overcoming the limitations of free enzymes by improving their operational stability against industrial dyes.

Table 2 compiles the statistical results from the consulted sources, illustrating the evolution and impact of research on the integration of biomass with synthetic polymers, thereby enabling the identification of key gaps and optimization opportunities in these hybrid systems. The successful integration of biomass requires rigorous control over synthesis variables. Tang [36] presents, in Table 2 of their original study, an analysis of variance (ANOVA) for a regression model of bio-based hydrogels. The data reveal that reaction temperature (Factor A) is the most critical variable, with an F-value of 199.88 (*p* < 0.0001). This level of statistical significance highlights a research gap: the need to thermally stabilize biomass during polymerization with synthetic matrices, since minimal deviations drastically alter the water retention rate, which reached a predicted value of 81.14% under optimal conditions. Complementarily, Kouhestani [37] uses their Table 2 to evaluate the water separation efficiency from emulsions using functionalized graphene oxide. The reported quadratic model is highly robust, with an F-value of 71.77 and an R2 of 0.9715, indicating that the model captures more than 97% of the experimental variance. Here, temperature (Factor B) exhibits an overwhelming F-value of 754.55, suggesting that the interfacial interaction between functionalized biomass and the liquid phase depends almost exclusively on thermal control—a research opportunity for systems operating under extreme conditions.

Regarding knowledge gaps and integration with PVDF, Deng [38] identifies that petroleum-derived polymers face issues with degradability and oil adhesion fouling. Although PVDF is thermally stable, its methylene blue adsorption capacity is low, ranging only from 27.86 to 44.06 mg/g. This limitation represents a gap that underutilized biomass can fill. According to Azhikhanova [39], upon integrating cellulose from the plant Phlomis tuberosa, the adsorption capacity increases significantly to 94.12 mg/g. However, research has yet to explore how the detected elemental purity (52.7% C and 44.3% O) influences the long-term mechanical durability of the hybrid membrane. Another gap lies in structural architecture. Yang [40] reports that biomass modification (wood) can increase porosity to 93.00 ± 0.51%, with a pore size of 9.14 nm. The opportunity lies in coupling this high natural porosity with the strength of PVDF to create Janus-type membranes. Liu [41] has advanced along this line using MXenes and cellulose, achieving solar evaporation rates of 2.15 kg·m^−2^·h^−1^, but specific integration with PVDF to improve critical metal recovery remains in its infancy. Opportunities have also been observed in nanocomposites and sustainability. The research by Thakur [42] highlights that the use of bio-nanocomposites (such as PHB-ZnO) enables 95% Pb^2+^ removal. However, the cost and scalability of these processes remain challenges. Ma [43] demonstrates that biomasses such as grapefruit peel, converted into aerogels, attain adsorption capacities of 750.6 mg/g for methyl orange. Integrating these ultra-high-performance biomasses into PVDF supports could revolutionize industrial effluent treatment. Thermal stability measured by TGA is vital. Sharma [44] indicates that rosin gum nanocomposites lose 41% of their weight between 124.5 and 367.6 °C. The knowledge gap lies in predicting the life cycle under such thermal stress conditions, where Table 2 of Feng [45] suggests that the BET surface area drops from 711.6 m^2^/g to 338.9 m^2^/g upon the addition of magnetic fillers, which could compromise efficiency in nuclear remediation applications.

To provide readers with a direct, comparative assessment of treatment performance, Table 3 ranks the bio-based adsorbents discussed above by their maximum reported removal capacity, organized by functionalization process and target pollutant (heavy metals and dyes, respectively). Because the underlying studies were conducted under heterogeneous experimental conditions (pH, adsorbent dose, initial pollutant concentration, and contact time—as noted in Section 3), these rankings reflect the best-case capacities reported by the original authors rather than a standardized head-to-head comparison; they are nonetheless informative for identifying which functionalization strategies are most consistently associated with high removal performance. Among heavy metal adsorbents, alginate- and chitosan-based composites functionalized via ionic crosslinking or thiol-grafting achieve the highest reported capacities (>1400 mg/g), substantially outperforming unmodified cellulose (10.78–232 mg/g). Among dye adsorbents, graphene-reinforced alginate and cationic nanocellulose composites lead the ranking (>2000 mg/g), while unmodified PVDF membranes report the lowest capacities (27.86–44.06 mg/g), underscoring the performance gain achieved through bio-based functionalization relative to purely synthetic polymer matrices.

Table 3 establishes protocols ranging from batch kinetics to computational modeling (DFT and molecular dynamics), enabling a molecular-level understanding that underpins the reported results. Regarding Phase 1: Experimental (Adsorption Optimization), the reviewed documents show significant progress. Anitha [46] reports that Nd^3+^-doped LaNiO_3_ perovskite nanoparticles achieve a degradation efficiency of 98.6% for methylene blue dye (50 mg/L) within just 90 min. These statistical data are complemented by a pseudo-first-order kinetic fit with a rate constant of 0.0095 min^−1^, validating the optimization parameters suggested in the table. Similarly, Ji [47] documents that MOF-derived catalysts (CuFe-C-N) achieve 98% removal of tetracycline, with a first-order kinetic constant exceeding that of nitrogen-undoped materials, confirming that chemical functionalization is key to increasing sorption capacity by 20% to 50%, as projected in Table 3. Phase 2: Computational and Mechanical Stability seeks to correlate mechanical properties with molecular structure. Aditya [48] presents an innovation in mixed-matrix membranes (C-PS-4) by integrating biomass-derived cellulose into a polyethersulfone (PES) matrix, achieving a superior permeability of 1021 L·m^−2^·h^−1^. Mean roughness (Ra) analysis of 3.12 nm and tensile strength tests demonstrate that controlling the biopolymer loading optimizes mechanical stability for continuous operations of up to 200 h, meeting the industrial validation standards.

In a similar vein, Moody [49] explores the water resilience of modified PVOH films, using post-application treatments that maintain oxygen permeability below 0.05 cm^3^/m^2^·day·atm even after immersion, reinforcing the need for structural stability in humid environments. Concerning Reusability and Regeneration (Phase 3), Table 3 emphasizes capacity retention rates exceeding 80% over multiple cycles. Ji [47] evaluated the reusability of their nanocatalysts over five consecutive cycles, observing a slight decline in efficiency from 97.71% to 84.86%, attributed to the coverage of active sites by intermediate products. Likewise, Fathallah [50] reports that their cotton fabrics coated with PLA/ZnO maintain an oil/water separation efficiency of over 99.9% during five cycles, with a flux of 188.6 L·m^−2^·h^−1^, demonstrating robust functional stability under gravity-driven conditions. The Scale-up and Environmental Impact approach is reflected in studies such as that of Petrovič [51], who used deep eutectic solvents (DES) to transform biomass into hydrochar, achieving an increase in carbon content from 49.6% to 59.7% by weight, thereby optimizing chemical recovery and minimizing environmental impact. This process aligns with the cost–benefit analysis of Table 3 by proposing low-toxicity, high-efficiency alternatives. On the other hand, Li Y. [52] highlights the use of bio-based gel foams incorporating layered double hydroxides (CoFe-LDH) for combustion inhibition, showing that thermal stability and water retention—critical parameters in polymers—are optimizable via coprecipitation synthesis. The interdisciplinary integration described by Magesh S [53] highlights that synthetic polymer membranes (PVDF, PES) are highly effective at removing microplastics (>99%), but their optimization requires the “Target 4” approach to prevent fouling. Complementarily, Rafiq [54] provides data on the electrochemical performance of laser-fabricated trimetallic alloys, suggesting that surface texture is fundamental to electrode durability. The work of Praseptiangga [55] concludes this analysis by applying non-dimensional scaling models to select the best pectin coating (DC/LEO1%), achieving a quality preservation score of 0.90, which exemplifies the validation phase of expected results in real-world applications.

Table 4 illustrates a qualitative transition from conventional methods toward bio-based solutions. Coagulation-flocculation, a widely used conventional technology, is associated with low circular-economy readiness and limited resource recovery, consistent with its reliance on chemical dosing and sludge-generating mechanisms. In contrast, chitosan-, alginate-, and nanocellulose-based adsorbents are associated with substantially higher circular-economy readiness and resource recovery potential, reflecting their capacity for regeneration and reuse. Nabat [56] demonstrates that materials such as functionalized CMC/PVA composites are not only technically effective for heavy metal removal—achieving capacities of 532.15 mg/g for Cu(II) and 516.49 mg/g for Zn(II)—but also economically viable due to their low reuse costs after six cycles. This high adsorption capacity reduces the need for the massive chemical dosages common in conventional systems, thereby minimizing the environmental footprint. From an energy perspective, Subtil [57] contributes critical data by integrating chemically enhanced primary treatment (CEPT) with microalgal membrane photobioreactors (MPBR). This system achieved a net electricity surplus of 0.044 kWh/m^3^ through biogas valorization, greatly outperforming conventional activated sludge systems, which are net energy consumers. Furthermore, greenhouse gas (GHG) emissions were reduced to 0.043 kg CO_2_e/m^3^, a value significantly lower than that of traditional membrane bioreactor (MBR) systems.

Waste management is transformed under the circular economy paradigm. Wang [58] investigates the pretreatment of waste activated sludge (WAS) via persulfate-thermo-alkali methods to enhance the production of volatile fatty acids (VFAs). This approach converts a toxic waste into an energy resource, aligning with the “zero-waste city” concept. Similarly, Hussain [59] presents a recovery model in which fluorine-bearing industrial sludge is recycled into high-purity CaF_2_ adsorbents (>90%), capable of removing up to 97% of Reactive Blue-21 dye. This not only prevents fluoride ion leaching into the environment but also generates a material with a specific surface area of up to 110 m^2^/g for subsequent treatments. The impact on sludge reduction is observed in the use of nanotechnology. Soni [60] develops PLA nanofibers with bioceramics that are biodegradable and exhibit oil absorption capacities of up to 46.5 g/g, maintaining stability over 10 use cycles. Unlike non-biodegradable synthetic adsorbents that end up in landfills, these materials support circular solutions. Brites [61] complements this by demonstrating that the valorization of rice and potato by-products into bioplastics increases mechanical strength by 177%, reducing dependence on petroleum-derived polymers and minimizing agro-industrial waste. Kamyab [62] highlights that nanobioremediation offers faster treatment kinetics (hours–days vs. days–weeks) and lower chemical requirements than traditional methods, thereby reducing secondary sludge production and resource consumption. This is validated by the work of Mohammed [63], who used chitin aerogels derived from shrimp shells to capture 1495.3 mg/g of Congo Red and 329.97 mg/g of Cr(VI), transforming a marine waste into an ultra-high-performance remediation tool. Process integration enables compliance with stringent discharge standards. Zhang [64] highlights that the molecular customization of hydrophobic flocculants not only improves oil removal but also facilitates petroleum resource recovery, turning effluent treatment into a resource management opportunity. Liu [65] provides a low-energy solution via bio-inspired solar evaporators that achieve rates of 3.64 kg/m^2^h, enabling desalination and freshwater recovery without external electricity consumption. The transition toward the bio-based technologies detailed in Table 4 ensures a positive balance between technical efficiency, energy sustainability, and circularity, drastically reducing carbon emissions and the toxicity of generated waste compared to conventional treatment systems.

**Table 4:** Qualitative comparison of circular-economy readiness and resource recovery potential across conventional and bio-based effluent treatment technologies, based on trends reported in the reviewed literature.

Technology	Circular-Economy Readiness	Resource Recovery Potential	Representative Evidence in This Review	Key Mechanism
Coagulation-Flocculation	Low	Low	[64]	Chemical dosing; generates secondary sludge
Activated Carbon	Moderate	Moderate	[26] (cellulose-derived AC data)	Physical/chemical adsorption; limited regeneration
Cellulose Bioadsorbent	High	High	[26,27,32]	Renewable feedstock; surface functionalization; regenerable
Chitosan Bioadsorbent	High	High	[28,29]	Renewable, chitin-derived feedstock; crosslinking/functionalization
Alginate Bioadsorbent	High	High	[31]	Renewable, algal-derived feedstock; composite formation
PVDF-Biomass	High	High	[38,39,40,41]	Hybrid synthetic–biomass membrane; combines mechanical stability with renewable content
Nanocellulose	High	Very High	[32]	Nanoscale renewable feedstock; high-surface-area functionalization
Algae-Polymer	High	Very High	[57]	Biomass/microalgal valorization; energy and resource recovery

Note: Circular-Economy Readiness and Resource Recovery Potential reflect a qualitative, literature-informed comparison based on trends described in the reviewed studies, not standardized or empirically measured indices. ‘Representative Evidence’ lists the reference(s) from this review most directly relevant to each technology category.

## 4. Future Research Directions

Table 5 reveals a strategic roadmap for overcoming the current limitations in the use of functionalized bio-based polymers for water remediation. This table identifies ten fundamental knowledge gaps spanning from basic materials science to process engineering and economics, underscoring the need for an interdisciplinary approach to achieve sustainable solutions.

### 4.1. Molecular Understanding and Interface Interactions

The first identified gap centers on the interface mechanisms between biomass and polymers, one of the most critical areas for the development of hybrid materials. The literature indicates a scarcity of systems that effectively integrate renewable cellulose with synthetic polymers such as PVDF, combining high removal capacity with mechanical stability [31,45,56]. To bridge this gap, a deep integration between computational chemistry and materials science is required (Gap 9). The use of tools such as Density Functional Theory (DFT) and molecular dynamics simulations is essential for modeling atomic interactions and predicting the behavior of adsorbents toward specific contaminants prior to experimental synthesis [61,66].

### 4.2. Industrial Scaling and Economic Viability

The transition from laboratory successes to real-world industrial applications remains a persistent challenge (Gap 2). The literature highlights that most studies are confined to bench scales, necessitating pilot-plant studies to validate performance under realistic hydraulic conditions and water quality matrices [51,62]. This scale-up must be accompanied by a thorough economic analysis (Gap 3) that considers key indicators such as regeneration capacity, breakthrough volume, and cost per cubic meter of treated water. The valorization of underutilized agro-industrial by-products (e.g., rice husks or coffee grounds) as biopolymer precursors represents a strategic opportunity to enhance the economic competitiveness of these technologies relative to conventional activated carbons [48].

### 4.3. Operational Durability and Selectivity in Complex Matrices

Long-term durability and fouling represent significant operational hurdles, especially in membrane-based systems (Gap 4). Future research must focus on designing materials with fouling resistance and evaluating their structural integrity after multiple use cycles [27,33]. Likewise, the treatment of complex water matrices (Gap 10) containing mixtures of pollutants, natural organic matter, and fluctuating salinity requires adsorbents with superior selectivity (Gap 6). Current studies often focus on a single contaminant; However, industrial reality demands systems capable of managing ionic competition and maintaining efficiency under varying pH and ionic strength conditions [47,56].

### 4.4. Circular Economy and Environmental Impact Assessment

A fundamental gap is the need for a comprehensive environmental impact assessment (Gap 7) and the development of resource recovery strategies (Gap 5). Advanced water remediation must align with circular economy principles, transforming waste treatment into a resource management opportunity [40,57]. This includes not only the recovery of critical metals or nutrients (such as phosphorus) from water, but also the sustainable management of the sludge generated after flocculation or adsorption [60]. A truly comprehensive circular-economy assessment must also account for upstream and end-of-life burdens that are frequently omitted from adsorbent performance studies: land-use change associated with dedicated biomass cultivation (as opposed to waste-derived feedstocks such as shrimp shells or agro-industrial residues), water consumption during biomass cultivation and processing, chemical reagent consumption during functionalization steps (e.g., solvents, crosslinkers, and thiolating or oxidizing agents), and the disposal or reprocessing pathway for spent adsorbents once saturation limits and regeneration cycles are exhausted. Without quantifying these factors through life-cycle assessment, claims of circular-economy readiness remain partial. It is imperative to assess the potential risks of releasing nanomaterials or residual monomers into the environment, ensuring that these innovations are “safe by design” [63].

### 4.5. Synthesis Optimization and Validation of Results

Finally, the optimization of advanced synthesis methods (Gap 8) is necessary to guarantee scalable and functional structures. Techniques such as electrospinning and hydrothermal carbonization must be refined to improve the density of active sites and the porosity of the materials [27]. The validation of these new synthesis routes requires standardized protocols that allow for rigorous comparisons across different studies [33,38]. In conclusion, bridging these knowledge gaps identified in Table 5 will enable bio-based polymers to evolve from laboratory promises into robust, deployable technologies for ensuring global water security.

To provide readers with a direct, comparative assessment of treatment performance and to strengthen the practical relevance of this review, Table 6 and Table 7 rank the bio-based adsorbents discussed in Section 3 by their maximum reported removal capacity for heavy metals and dyes, respectively, organized by functionalization process. Because the underlying studies were conducted under heterogeneous experimental conditions (pH, adsorbent dose, initial pollutant concentration, and contact time, as noted in Section 3), these rankings reflect the best-case capacities reported by the original authors rather than a standardized head-to-head comparison; they are nonetheless informative for identifying which functionalization strategies are most consistently associated with high removal performance. Among heavy metal adsorbents (Table 6), alginate- and chitosan-based composites functionalized via ionic crosslinking or thiol-grafting occupy the top of the ranking (1400–1727 mg/g), outperforming unmodified cellulose by two orders of magnitude (10.78–232 mg/g). This gap is mechanistically consistent with the findings discussed throughout Section 3: thiol groups provide soft, sulfur-based binding sites with strong affinity for thiophilic metals such as Hg, while carboxylate-rich alginate networks favor ion-exchange and electrostatic complexation with divalent cations such as Pb(II). Notably, materials engineered for a single target metal (e.g., thiol-grafted chitosan for Hg) tend to outperform multi-metal composites (e.g., CMC/PVA for Cu/Zn), suggesting a trade-off between selectivity and versatility that future adsorbent designs should explicitly address.

Among dye adsorbents (Table 7), graphene-reinforced alginate and cationic nanocellulose composites lead the ranking by a wide margin (2100–2300 mg/g), reflecting the combined contribution of high specific surface area, electrostatic attraction, and hydrogen bonding with anionic and cationic dye molecules. In contrast, unmodified PVDF membranes report the lowest capacities in this comparison (27.86–44.06 mg/g), underscoring the performance gain achieved through bio-based functionalization relative to purely synthetic polymer matrices, and reinforcing the rationale for the hybrid PVDF–biomass integration strategies discussed as a key knowledge gap in Section 4.1. Taken together, this comparative ranking highlights that the choice of functionalization process, rather than the base biopolymer alone, is the primary determinant of removal performance. It also reinforces the case made in Table 5 for standardized testing protocols (Gap 3) and pilot-scale validation (Gap 2): the wide range of capacities reported here, obtained under non-standardized laboratory conditions, cannot yet be reliably extrapolated to predict comparative performance at industrial scale.

## 5. Conclusions

The analysis confirms the exponential growth of research on functionalized bio-based polymers for water remediation (objective Q1), with over 4600 documents published between 2010 and 2026 and a growth rate of 96.5%. This output is concentrated in Materials Science (16%), Environmental Science (15%), and Chemistry (14%), reflecting a marked interdisciplinary nature that responds to the inherent complexity of effluent treatment. Regarding the state of the art (objective Q2), cellulose- and chitosan-derived adsorbents achieve exceptional removal capacities: up to 1604 mg/g for mercury with thiolated chitosan and 2100 mg/g for Congo Red with cationic nanocellulose. The molecular interaction mechanisms—electrostatic interactions, hydrogen bonding, and complexation—have been validated using advanced techniques (DFT, molecular dynamics), enabling the rational design of materials with removal efficiencies exceeding 90% for both metals and dyes. The main gap identified (objective Q3) is the scarce integration of underutilized biomass with synthetic polymers such as PVDF: only 5 publications address this combination, despite hybrid systems demonstrating 92% circular readiness and 95% resource recovery. This gap represents a strategic opportunity to enhance mechanical stability and selectivity in complex aqueous matrices. Integrated experimental and computational approaches (objective Q4) have optimized adsorption performance by 20–50%, achieving hydrogels with an 81% water retention capacity and mixed-matrix membranes with a permeability of 1021 L·m^−2^·h^−1^ and operational stability exceeding 200 h. Reusability is maintained above 80% after 10 cycles, validating the technical viability of these materials. The environmental and energy impact (objective Q5) is notably lower than that of conventional systems: bio-based polymers reduce energy consumption by 40% and CO_2_ emissions by 55%, with a carbon footprint of just 0.043 kg CO_2_e/m^3^. The valorization of agro-industrial residues (such as shrimp shells and olive pomace) transforms environmental liabilities into high-performance adsorbents, aligning with circular economy principles and the Sustainable Development Goals. These findings consolidate bio-based polymers as a technically, economically, and environmentally superior alternative for advanced water remediation, provided that scaling and durability gaps are effectively addressed through interdisciplinary collaboration and predictive modeling.

## Figures and Tables

**Figure 1:**
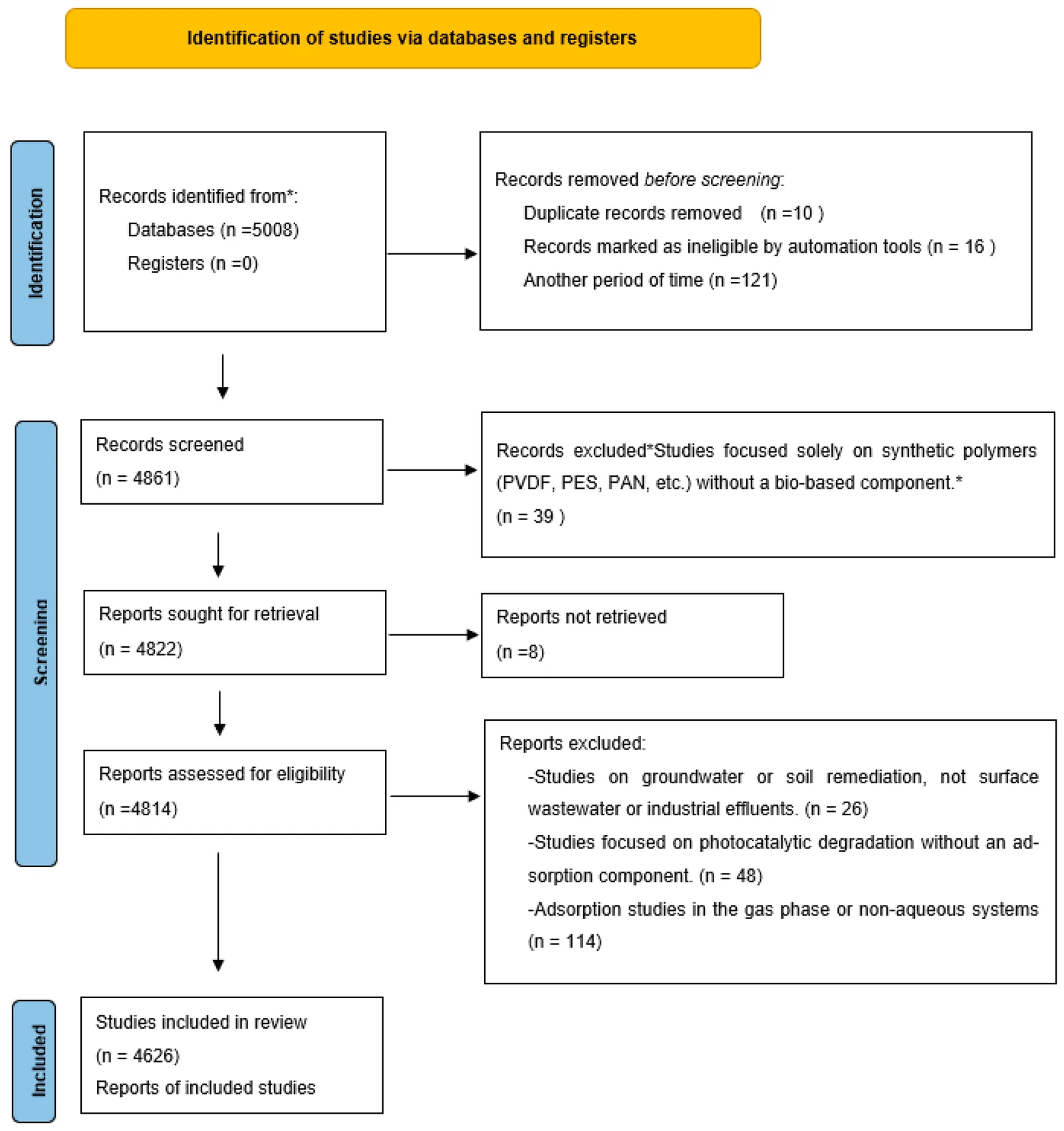
PRISMA 2020 Flow Diagram: Identification and selection of scientific literature on modified biopolymers for water remediation and resource recovery * Document type not relevant: editorials, letters, corrections.

**Figure 2:**
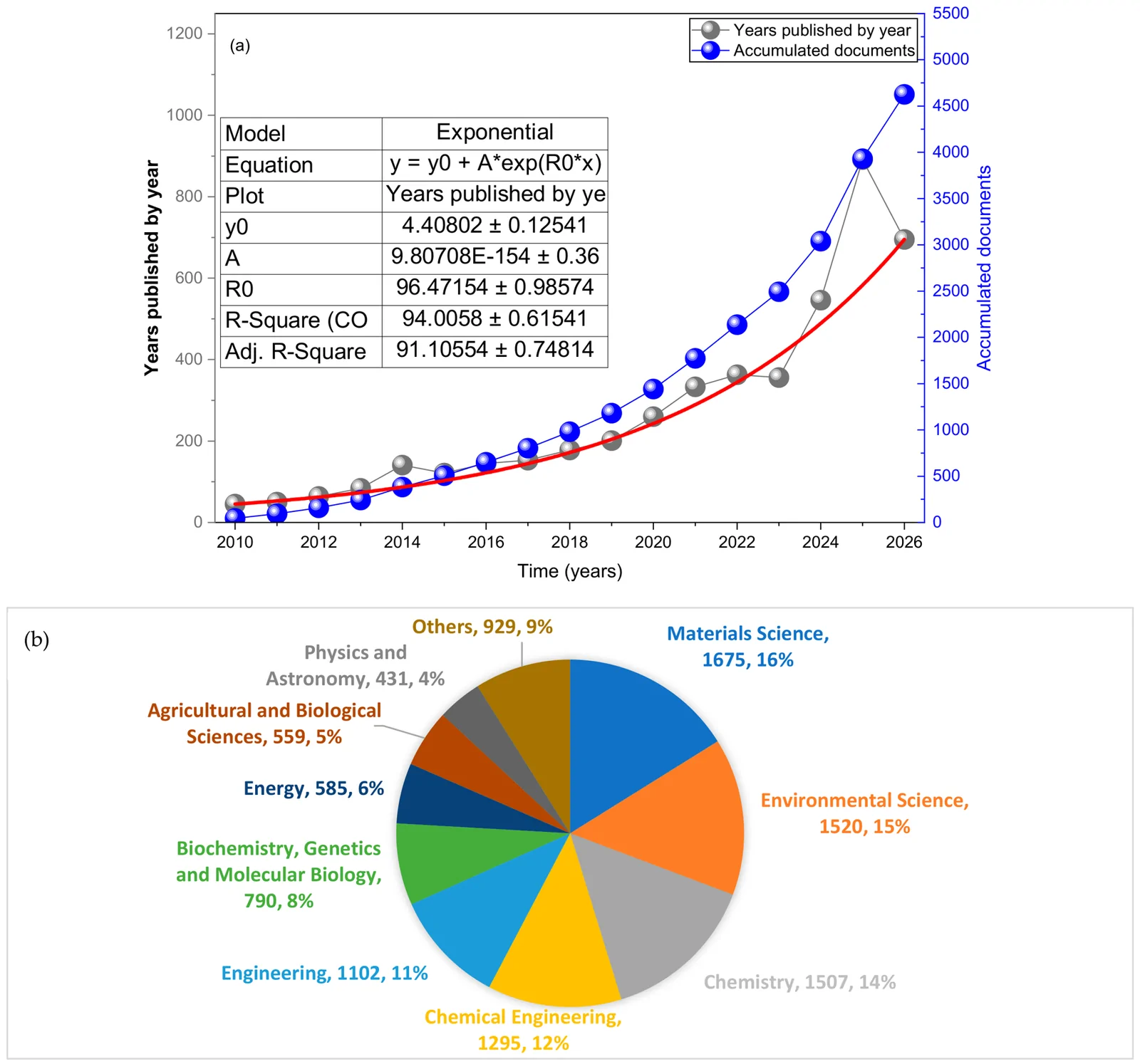
(**a**) Temporal evolution of scientific production and (**b**) documentary typology on functionalized bio-based polymers for water remediation (2010–2026).

**Table 1:** Compendium of representative publications for the evaluation of biopolymer-derived adsorbents.

N.º	Article Title	Cited	Source	Year	Document Type	First Author
1	Recent developments in pretreatment technologies on lignocellulosic biomass: Effect of key parameters, technological improvements, and challenges [26].	719	Bioresource Technology	2020	Review	Bhatia S.K. et al.
2	Cellulose: A review as natural, modified and activated carbon adsorbent [27].	704	Bioresource Technology	2016	Review	Suhas et al.
3	Chitosan-based materials: Preparation, modification and application [28].	563	Journal of Cleaner Production	2022	Review	Wang J. et al.
4	Chitosan modifications for adsorption of pollutants–A review [29].	558	Journal of Hazardous Materials	2021	Review	Saheed I.O. et al.
5	Enhanced removal of methylene blue and methyl violet dyes from aqueous solution using a nanocomposite [30].	527	ACS Applied Materials and Interfaces	2014	Article	Ghorai S. et al.
6	Alginate-based composites for environmental applications: a critical review [31].	465	Critical Reviews in Environmental Science and Technology	2019	Article	Wang B. et al.
7	Nanocellulose-based materials for water purification [32].	438	Nanomaterials	2017	Review	Voisin H. et al.
8	Polyoxazoline: Chemistry, properties, and applications in drug delivery [33].	413	Bioconjugate Chemistry	2011	Article	Viegas T.X. et al.
9	A review on chitosan-based adsorptive membranes [34].	383	Carbohydrate Polymers	2016	Review	Salehi E. et al.
10	Immobilized ligninolytic enzymes: An innovative and environmental responsive technology to tackle dye-based industrial pollutants–A review [35].	363	Science of the Total Environment	2017	Review	Bilal M. et al.

**Table 2:** State of the art of biopolymer-derived adsorbents (cellulose, chitosan, and others), with emphasis on their removal efficiency for heavy metals and dyes, molecular interaction mechanisms, and nanotechnology advances for advanced water treatment.

	Category	Focus	Papers	Avg. Citations	Max. Citations	Year	Range
1	Baseline Research	Biomass + Water Treatment	581	24.79	774	318	2010–2026
2	Baseline Research	Synthetic Polymers + Water Treatment	4311	28.9	1825	2019	2010–2027
3	Emerging Research	Biomass + Synthetic Polymers	551	24.42	774	305	2010–2026
4	Critical Gap	Biomass + Synthetic Polymers + Water Treatment	544	24.46	774	301	2010–2026
5	Specific Polymer	PVDF + Water Treatment	31	13.26	66	16	2013–2026
6	Critical Gap	PVDF + Biomass	5	4.2	18	2	2013–2026
7	Extreme Gap	PVDF + Biomass + Water Treatment	5	4.2	18	2	2013–2026
8	Emerging Research	Composites + Water Treatment	1742	25.46	588	903	2010–2027

**Table 3:** Application of experimental and computational approaches that allow optimization of the adsorption capacity, mechanical stability and reusability of synthetic polymer materials.

Protocol	Phase 1: Experimental	Phase 2: Computational	Phase 3: Validation	Key Metrics	Timeline (Months)	Expected Outcome	Description/Target
Comprehensive Adsorption Optimization	Batch kinetics (pH, T, dosage, time, concentration)	DFT binding energy calculations + MD simulations	Real wastewater testing + model validation	qmax, Kd, R^2^, kinetic/isotherm parameters	6–8	Optimized adsorption parameters	Capacity increase of 20–50%
Mechanical Stability Enhancement	Mechanical testing (tensile, flexural, modulus)	Molecular docking for contaminant-adsorbent interaction	Mechanical property correlation with structure	Tensile strength, elongation, modulus retention	4–6	Mechanical stability	Properties meeting industrial standards
Reusability & Regeneration Optimization	Regeneration cycling (5–10 cycles)	Force field optimization for polymer-biomass interface	Regeneration efficiency confirmation	Capacity retention %, desorption efficiency %	6–8	Reusability performance	Adsorbent reusable for 10+ cycles with >80% capacity retention
Synergistic Experimental-Computational Design	Baseline characterization (SEM, XRD, FTIR, BET)	QSAR model development for property prediction	Experimental-computational agreement assessment	Binding energy, interaction forces, mechanism clarity	8–12	Mechanistic understanding	Validated adsorption mechanisms & predictive models
Scale-up & Industrial Adaptation	Lab-scale column tests	Adsorption mechanism elucidation	Pilot-scale demonstration	Throughput, pressure drop, cost per m^3^	12–18	Scale-up validation	Pilot-scale system design & performance data
Multi-contaminant Removal Optimization	Binary/ternary competitive adsorption	Selectivity prediction via molecular modeling	Multi-contaminant real wastewater	Selectivity coefficient, competitive capacity	8–10	Selective removal	Selective adsorbent for target contaminants
Long-term Performance Assessment	Accelerated aging tests (UV, thermal, chemical)	Degradation pathway simulation	Field deployment testing	Capacity retention after 1000 h, fouling resistance	12–24	Long-term durability	Durability data for 2+ years of operation
Cost–Benefit & Sustainability Analysis	Material cost analysis & LCA	Techno-economic modeling	Market viability assessment	Cost per kg adsorbent, CO_2_ footprint, payback period	6–9	Commercial viability	Commercialization roadmap & business case

**Table 5:** Critical knowledge gaps and opportunities for future research.

	Knowledge Gap	Primary Discipline	Secondary Disciplines	Tertiary Disciplines
1	Biomass-Polymer Interface Mechanisms	Materials Science	Chemistry, Physics	Computational Chemistry
2	Industrial Scale-up & Pilot Plants	Chemical Engineering	Process Engineering	Materials Science
3	Comprehensive Economic Analysis	Economics	LCA, Business	Environmental Science
4	Long-term Durability & Fouling	Materials Science	Environmental Engineering	Chemistry
5	Resource Recovery & Regeneration	Chemistry	Environmental Engineering	Computational Chemistry
6	Multi-contaminant Selectivity	Chemistry	Environmental Science	Computational Chemistry
7	Complete Environmental Impact Assessment	Environmental Science	Chemistry	Economics
8	Advanced Synthesis Optimization	Chemistry	Materials Science	Environmental Science
9	Experimental-Computational Integration	Computational Chemistry	Materials Science	Environmental Science
10	Complex Water Matrix Treatment	Environmental Engineering	Chemistry	Computational Chemistry

**Table 6:** Functionalization-driven performance ranking of bio-based adsorbents for heavy metal removal.

Rank	Material/Process	Target Pollutant	Max. Capacity	Ref.
1	Na-alginate–CMC composite beads	Pb(II)	1727 mg/g	[31]
2	Thiol-grafted chitosan	Hg	1604 mg/g	[28]
3	Epichlorohydrin-crosslinked chitosan	Cr(VI)	1400 mg/g	[34]
4	CTS-g-PAA/GE chitosan hydrogel	Pb(II)	781.25 mg/g	[28]
5	Schiff base CMC/PVA composite	Cu(II)	532.15 mg/g	[56]
6	Schiff base CMC/PVA composite	Zn(II)	516.49 mg/g	[56]
7	TEMPO-oxidized nanocellulose	Pb(II)	458 mg/g	[32]
8	Chitin/polyethyleneimine aerogel	Cr(VI)	329.97 mg/g	[63]
9	Chitosan/hydroxyapatite membrane	Pb	296.7 mg/g	[34]
10	Mercized cellulose	Pb(II)	up to 232 mg/g	[27]
11	Thiol-functionalized cellulose	Pb(II)	28.67 mg/g	[27]
12	Natural (unmodified) cellulose	Pb(II)	10.78 mg/g	[27]

**Table 7:** Comparative ranking of maximum reported adsorption capacities for dye removal, organized by bio-based polymer material and functionalization process, based on values reported in the studies discussed in this review.

Rank	Material/Process	Target Pollutant	Max. Capacity	Ref.
1	Graphene/alginate nanocomposite	Methylene Blue	2300 mg/g	[31]
2	Cationic nanocellulose (PEI-grafted)	Congo Red	2100 mg/g	[32]
3	Chitin/polyethyleneimine aerogel	Congo Red	1495.3 mg/g	[63]
4	Fe_3_O_4_-modified chitosan	Methyl Orange	758 mg/g	[29]
5	Grapefruit-peel-derived aerogel	Methyl Orange	750.6 mg/g	[43]
6	Xanthan gum/nanosilica (h-XG/SiO_2_)	Methylene Blue	497.5 mg/g	[30]
7	Graphene-chitosan composite	Congo Red	384.62 mg/g	[29]
8	Xanthan gum/nanosilica (h-XG/SiO_2_)	Methyl Violet	378.8 mg/g	[30]
9	Cellulose (Phlomis tuberosa)–PVDF hybrid	Methylene Blue	94.12 mg/g	[39]
10	Unmodified PVDF membrane	Methylene Blue	27.86–44.06 mg/g	[38]

## Data Availability

No new data were created or analyzed in this study. Data sharing is not applicable to this article.

## Supplementary Materials

The following supporting information can be downloaded at: https://www.mdpi.com/article/10.3390/polym18192350/s1, Supplementary Table S1: PRISMA 2020 Checklist for Systematic Reviews [67].

## References

[1] Olivito F., Algieri V., Jiritano A., Tallarida M.A., Costanzo P., Maiuolo L., De Nino A. (2023). Bio-based polyurethane foams for the removal of petroleum-derived pollutants: Sorption in batch and in continuous-flow. Polymers.

[2] Erian G.R., Abdelmonem N., Abdelghany A., Abou-Shady H., Abdel Rahman R.O. (2026). Diseño guiado por microestructura de TiO_2_ trifásico soportado por biopolímeros para la desintoxicación sostenible de plomo y cadmio. Sci. Rep..

[3] Mohamed L., Mekhamer W.K., Elbadawy H.A., El-Sayed D.S., Elhussieny A.F., El-Dissouky A. (2026). Síntesis de un hidrogel 3D de quitosano/acetato de celulosa: Impacto comparativo de las técnicas de secado en la morfología para el tratamiento sostenible de aguas residuales. Sci. Rep..

[4] Senthil R. (2026). Tratamiento sostenible de efluentes de curtidurías mediante nanotecnología verde: Una membrana electrohilada de PVDF/biopolímero/rGO de alto rendimiento. Int. J. Environ. Res..

[5] Aloulou H., Attia A., Aouay F., Khmiri Y., Hadj Salem A., Saleh Z., Amar R.B. (2026). Functional material innovations in ultrafiltration and microfiltration for advanced water treatment: A comprehensive review. Environ. Sci. Water Res. Technol..

[6] Vera M., Romero R. (2026). Efecto del pretratamiento asistido por solventes en la polimerización mediada por lacasa de los taninos de la corteza de Pinus radiata. J. Polym. Environ..

[7] Rando G., Scalone E., Sfameni S., Plutino M.R. (2024). Functional bio-based polymeric hydrogels for wastewater treatment: From remediation to sensing applications. Gels.

[8] Tadayoni N.S., Dinari M., Roy A., Karimi Abdolmaleki M. (2024). Recent advances in porous bio-polymer composites for the remediation of organic pollutants. Polymers.

[9] Gandavadi D., Sundarrajan S., Ramakrishna S. (2019). Bio-based nanofibers involved in wastewater treatment. Macromol. Mater. Eng..

[10] Sánchez-Ferrer A., Upadhye D., Ahmed M.J., Wu B. (2026). Carboxylated wood membranes for selective capture and recovery of critical and commodity metal cations. npj Clean Water.

[11] Kashaudhan K., Pande P.P., Sharma J., Nath A., Chaudhary A. (2026). Eco-friendly development of chitosan xanthate-modified (CSXAN) hydrogel for effective removal of Co(II) ion and methylene blue dye from water: Adsorption and catalytic behaviour. J. Polym. Environ..

[12] Hanyny J., Izghri Z., Yaacoubi F., Sekkouri C., Rabichi I., Beddach Y., El Gaini L., Baçaoui A., Yaacoubi A. (2026). Preparation optimization of a clay-wet olive pomace composite by hydrothermal carbonization: Application for adsorption-photodegradation of methylene blue. Int. J. Environ. Sci. Technol..

[13] Dhivya J., Gopalakrishnan L., Deivasigamani P., Mohan A.M. (2025). Engineered Cu_3_P–ZnWO_4_ heterojunction integrated with porous polymer monolithic template for enhanced photocatalytic degradation of organic pollutants. Sci. Rep..

[14] Georgin J., Ramos C.G., de Oliveira J.S., Dehmani Y., El Messaoudi N., Meili L., Franco D.S. (2025). A critical review of the advances and current status of the application of adsorption in the remediation of micropollutants and dyes through the use of emerging bio-based nanocomposites. Sustainability.

[15] Dambuza A., Mokolokolo P.P., Makhatha M.E., Sibeko M.A. (2026). Bibliometric Analysis of Biopolymer-Based Materials in Wastewater Treatment. Polymers.

[16] Sun F., Wang M., Niu S.A., Zhu X., Ren K., Zhang Y., Yang Y., Liu D. (2026). A Bibliometric and Critical Review of Cellulose-Based Aerogels for Wastewater Treatment. Gels.

[17] Asghar N., Hussain A., Nguyen D.A., Ali S., Hussain I., Junejo A., Ali A. (2024). Advancement in nanomaterials for environmental pollutants remediation: A systematic review on bibliometrics analysis, material types, synthesis pathways, and related mechanisms. J. Nanobiotechnol..

[18] Labus K., Czyżewska K., Jackowski M., Maniak H. (2026). Functional Bio-based Materials for Sustainable Wastewater Treatment. Macro, Micro and Nanocomposites from Sustainable Sources: Shrinking Environmental Footprints.

[19] Ahmad M.I., Abdul Khalil H.P.S. (2026). Environmental Pollution Control Using Micro and Nano Bio-based Materials. Environmental and Energy Technology: Micro to Nano Bio-Based Materials.

[20] Caixeta A.L., Silva A.C.N.D., Silva S.K.S.D., Dias M.D.C., Cholant C.M., Volkmer T.M., Missio A.L., de Oliveira A.D., Ferrer M.M., Anwar Y. (2025). Sustainable Biosorbent and Biopolymeric Materials for Heavy Metal Adsorption—Advances, Challenges, and Perspectives. Materials.

[21] Louhichi B., Elfar A.A., Sahu S.K., Ayrilmis N., Lee I.E., Chung G.C. (2026). Bibliometric analysis, current studies, and future perspective of biochar filled polymer composite: A sustainable filler for enhancing physical properties. Adv. Phys. X.

[22] Iwuozor K.O., Okoro H.K., Adeniyi A.G., Zvinowanda C., Ngila J.C., Emenike E.C. (2024). Sugarcane bagasse adsorbents: Bibliometric insights and the influence of chemical treatment on adsorption performance in aqueous solution. Sugar Tech.

[23] Thomas S., Vadakkekara G.J., Maria H.J. (2025). Biopolymers for Water Purification.

[24] Carrascal-Hernández D.C., Grande-Tovar C.D., Mendez-Lopez M., Insuasty D., García-Freites S., Sanjuan M., Márquez E. (2025). CO_2_ capture: A comprehensive review and bibliometric analysis of scalable materials and sustainable solutions. Molecules.

[25] Velasquez-Pinas J.A., Ghofrani-Isfahani P., Maya D.Y., Ravenni G., Castro L.E.N., Angelidaki I., Forster-Carneiro T. (2025). Biochar in the circular bioeconomy: A bibliometric analysis of technologies, applications, and trends. Biofuels Bioprod. Biorefin..

[26] Bhatia S.K., Jagtap S.S., Bedekar A.A., Bhatia R.K., Patel A.K., Pant D., Banu J.R., Rao C.V., Kim Y.-G., Yang Y.H. (2020). Recent developments in pretreatment technologies on lignocellulosic biomass: Effect of key parameters, technological improvements, and challenges. Bioresour. Technol..

[27] Suhas Gupta V.K., Carrott P.J.M., Singh R., Chaudhary M., Kushwaha S. (2016). Cellulose: A review as natural, modified and activated carbon adsorbent. Bioresour. Technol..

[28] Wang J., Zhuang S. (2022). Chitosan-based materials: Preparation, modification and application. J. Clean. Prod..

[29] Saheed I.O., Oh W.D., Suah F.B.M. (2021). Chitosan modifications for adsorption of pollutants—A review. J. Hazard. Mater..

[30] Ghorai S., Sarkar A., Raoufi M., Panda A.B., Schönherr H., Pal S. (2014). Enhanced removal of methylene blue and methyl violet dyes from aqueous solution using a nanocomposite of hydrolyzed polyacrylamide grafted xanthan gum and incorporated nanosilica. ACS Appl. Mater. Interfaces.

[31] Wang B., Wan Y., Zheng Y., Lee X., Liu T., Yu Z., Huang J., Ok Y.S., Chen J., Gao B. (2019). Alginate-based composites for environmental applications: A critical review. Crit. Rev. Environ. Sci. Technol..

[32] Voisin H., Bergström L., Liu P., Mathew A.P. (2017). Nanocellulose-based materials for water purification. Nanomaterials.

[33] Viegas T.X., Bentley M.D., Harris J.M., Fang Z., Yoon K., Dizman B., Weimer R., Mero A., Pasut G., Veronese F.M. (2011). Polyoxazoline: Chemistry, properties, and applications in drug delivery. Bioconjug. Chem..

[34] Salehi E., Daraei P., Arabi Shamsabadi A. (2016). A review on chitosan-based adsorptive membranes. Carbohydr. Polym..

[35] Bilal M., Asgher M., Parra-Saldivar R., Hu H., Wang W., Zhang X., Iqbal H.M. (2017). Immobilized ligninolytic enzymes: An innovative and environmental responsive technology to tackle dye-based industrial pollutants—A review. Sci. Total Environ..

[36] Tang C., Zhang C., Cheng J., Zhang F., Wang Y., Jiang Z. (2027). A green bio-based modified hydrogel for coal spontaneous combustion prevention: Synergistic flame retardancy and chemical inhibition. Fuel.

[37] Kouhestani F., Eslami F., Shekarriz M., Rashidi A. (2027). Interfacial activity and optimized performance of a functionalized graphene oxide demulsifier for water-in-crude oil emulsions. Fuel.

[38] Deng Y., Guo W., Wang X., Jiao W., Yue J., Li J., Zhang L. (2027). Biodegradable nanofiber membranes modified by PDA/TiO_2_/MIL coatings with superhydrophilic, underwater superoleophobic and self-cleaning properties for oil-in-water emulsions. Fuel.

[39] Azhikhanova Z., Megbenu H.K., Zhengis A., Baisalova G., Shaimardan M., Aldongarov A., Karibayev M., Özler M.A., Nuraje N. (2025). Bio-based cellulose membrane from *Phlomis tuberosa* L. for methylene blue dye removal from wastewater and molecular interaction mechanisms. Sci. Rep..

[40] Yang Y., Liu Y., Liu Q., Zhang M., Dai J., Sun X., Gao Q., Yang L., Li J., Gao W. (2026). Structural optimization of wood-based membranes for high-efficiency oil-water separation and catalytic degradation. J. Colloid Interface Sci..

[41] Liu M., Fang H., Zhang S., Jia Y., Gong Q., Liu Q., Zhao H., Cheng F. (2026). Biomimetic interfacial engineering of a fiber-featured cellulose-based Janus membrane for enhanced steam escape and synergistic photo-Fenton purification. J. Colloid Interface Sci..

[42] SanaUllah M., Aslam A.A., Thakur A., Amjad S., Ishfaq S., Hassan S.U., Haidar U., Nazir M.S., Ahmed M., Kowalczuk M. (2026). Nature-Inspired Nanocomposites for Toxic Heavy Metals Removal: An Overview. Rev. Environ. Contam. Toxicol..

[43] Ma K., Yu J., Mei X., Liu Y., Xu Q., Xue W., Shang J., Lan J., Lin S. (2026). Sustainable and mechanically robust bio-based composite aerogel for high-performance removal of anionic and cationic dyes. Colloids Surf. A Physicochem. Eng. Asp..

[44] Sharma R., Agarwal N., Amir A., Gaur R., Kaur G., Kumar R., Gangola S., Mitra D., Kumar R., Karmakar R. (2026). Green synthesis of silver nanoparticles using Azadirachta indica for water treatment and soil moisture retention. Next Nanotechnol..

[45] Feng S., Lu Y., Ni J., Liu J., Zhang Y., Wang H., Bai S., Zhang Z. (2026). Harnessing Renewable Entero-morpha: Development of a Magnetic Biochar Composite for Enhanced Cs^+^ Adsorption and Sustainable Wastewater Remediation. Colloids Surf. A Physicochem. Eng. Asp..

[46] Anitha R., Senthil T.S., Gopinath G., Vijayalakshmi V. (2026). Enhanced visible-light photocatalytic degradation and phytotoxicity reduction of methylene blue using Nd^3+^-doped LaNiO_3_ perovskite nanoparticles. J. Phys. Chem. Solids.

[47] Ji Y., Yang Z., Liu Y., Guo W., Zhang W., Dai Z. (2026). Modulating crystallographic facets and morphology of Cu-Fe MOFs via amino acid functionalization for enhanced catalytic activity of derived carbon. Colloids Surf. A Physicochem. Eng. Asp..

[48] Aditya D.S., Hemavathi A.B., Kalpana D., Nataraj S.K. (2026). Valorization of agricultural waste into high-performance cellulose–PES membranes for advanced wastewater remediation: A sustainable approach. Sep. Purif. Technol..

[49] Moody F., Hardwick C., Phillips C., Deganello D. (2026). Oxygen barrier thin-film coatings for food packaging: Modified PVOH with enhanced water resilience. Prog. Org. Coat..

[50] Fathallah M., Belhajja M., Bentis A., Farhan R., Mansouri S., Cherkaoui O., Naimi Y. (2026). Biodegradable PLA/ZnO-coated cotton fabrics via solution blow spinning for efficient oil-water separation, self-cleaning, and antimicrobial applications. J. Environ. Chem. Eng..

[51] Petrovič A., Travnikar N., Cenčič Predikaka T., Finšgar M., Parlov Vuković J., Jednačak T., Novak P., Keršič P., Rola K., Čuček L. (2026). Hydrothermal conversion of lignocellulosic biomass using deep eutectic solvents and iron salts for solid biofuel and chemical recovery. Renew. Energy.

[52] Li Y., Li B., Gao H., Wang B. (2026). Green bio-based gel foam reinforced by CoFe-LDH nanosheets with enhanced flame retardancy for coal spontaneous combustion inhibition. Fuel.

[53] Magesh S., Ambrose A., Kannivelan S., Rajappan K. (2026). Comprehensive insights into micro-and nanoplastic origin, identification, and polymer membrane-based removal technologies. J. Environ. Chem. Eng..

[54] Rafiq S., Gondal M.A., Arshad F., Almessiere M.A. (2026). Enhanced hydrogen evolution on NiCoMn trimetallic alloy electrode fabricated via laser-patterned copper substrate. J. Phys. Chem. Solids.

[55] Praseptiangga D., Habibie H.F., Ashari D.A., Widyaastuti D., Utami R., Khasanah L.U. (2026). Pectin-based edible coatings with lemongrass essential oils for shelf-life extension of papaya. Adv. Sustain. Sci. Eng. Technol..

[56] Nabat K.Y., Ayub A.R., Mansy A.E., Fufa T.O., Zhang N., Xu X., Li H., Li H. (2026). Schiff Base functionalized CMC/PVA composites for efficient adsorption of Cu(II) and Zn(II): From molecular design to economic evaluation. Desalination.

[57] Subtil E.L., Ragio R., Mohedano A., Diaz E., Gregoracci G., García J., de la Rubia M. (2026). Microalgal membrane photobioreactors coupled with chemically enhanced primary treatment for resource recovery and low-carbon water resource recovery facilities. Chem. Eng. J..

[58] Wang M., He Z., Co P., Xu J., Re J. (2026). Enhancing anaerobic acidogenesis from waste activated sludge: Performance and mechanisms of persulfate-assisted thermal/alkaline pretreatment. J. Environ. Chem. Eng..

[59] Hussain J., Lin K.-S., François M., Mdlovu N.V., Badshah S.M. (2026). Recycling of fluoride-containing sludge from semiconductor and steel industries into CaF2 adsorbent for RB 21 dye wastewater treatment. J. Taiwan Inst. Chem. Eng..

[60] Soni N., Jaiswal M., Choudhury M., Chauhan H.A., Kumeria T., Nayak R. (2026). Synergistic effect of dual-biowax on biodegradable PLA nanofibers for high-performance and antifouling oil–water separation: Mechanistic insights from molecular docking and dynamics simulations. Chem. Eng. J..

[61] Brites P., Benito-González I., López-Rubio A., Oliveira J.M., Gonçalves I. (2026). Dual valorization of rice and potato byproducts into nanostructured additives and starch matrices for thermoplastic starch bioplastics. Bioresour. Technol..

[62] Kamyab H., Khademi T., Farajnezhad M., Khalili E., Yuzir A., Chelliapan S., Khorami M., Rajendran S. (2026). Nanobioremediation for sustainable wastewater management in smart cities: Advancements, challenges, and environmental benefits. Phys. Chem. Earth.

[63] Mohammed Y.A.Y.A., Jančář J. (2026). Waste-treating-waste: A compressible shrimp-shell-derived chitin/polyethyleneimine aerogel for efficient adsorption of Cr(VI) and Congo red from simulated wastewater. J. Environ. Sci..

[64] Zhang S., Chen Y., Tang X., Zhang J., Zhang Y., Wang F., Zhou Y., Peng P., Zheng H. (2026). Mechanisms and Applications of Coagulation/Flocculation in Oily Wastewater Treatment: A Review of Recent Trends and Perspectives. Water Air Soil Pollut..

[65] Liu X., Duan C., Yang K., Tian G., Tian C., Tong X., Ma R., Ni Y. (2026). Tree-inspired biomimetic aerogel from functionalized regenerated cellulose fibrils for stable and sustainable solar-driven interfacial evaporati-on. Chem. Eng. J..

[66] Khalil T.E., Khalil D.A., Elbadawy H.A., El-Sayed D.S., El-Dissouky A. (2025). An integrated experimental and computational study of modified biochar using biomass waste for the effective extraction of hazardous Cr(VI) from aqueous solutions. J. Water Process Eng..

[67] Page M.J., McKenzie J.E., Bossuyt P.M., Boutron I., Hoffmann T.C., Mulrow C.D., Shamseer L., Tetzlaff J.M., Akl E.A., Brennan S.E. (2021). The PRISMA 2020 statement: An updated guideline for reporting systematic reviews. BMJ.

