# Supplementary material for: Sustainable Bio-Based Polymer Adsorbents for Water Remediation: A Systematic Review of Functionalization Strategies, Performance Enhancement, and Circular Economy Integration
Source: Polymers (Basel). 2026 Sep 26;18(19):2350. doi: 10.3390/polym18192350 (PMC13667133; doi:10.3390/polym18192350)
Supplement: Supplementary file 1 [file polymers-18-02350-s001.zip › polymers-4577017-supplementary.pdf]

PRISMA 2020 Checklist

Section and Topic	Item #	Checklist item	Location where item is reported
**TITLE**			
Title	1	Identify the report as a systematic review.	Title: "Sustainable Bio-Based Polymer Adsorbents for Water Remediation: A Systematic Review of Functionalization Strategies, Performance Enhancement, and Circular Economy Integration"
**ABSTRACT**			
Abstract	2	See the PRISMA 2020 for Abstracts checklist.	Abstract section (page 1) – includes background, objectives, methods (PRISMA, databases, tools), key results (growth rate, adsorption capacities, gaps), and conclusions.
**INTRODUCTION**			
Rationale	3	Describe the rationale for the review in the context of existing knowledge.	Introduction, paragraphs 1–3 (pages 1–2) – describes the global water crisis, industrial pollution (dyes and heavy metals), limitations of conventional methods, and the potential of bio-based polymers as sustainable alternatives.
Objectives	4	Provide an explicit statement of the objective(s) or question(s) the review addresses.	Introduction, final paragraph (page 3) – states the overall objective and presents five specific research questions (Q1–Q5).
**METHODS**			
Eligibility criteria	5	Specify the inclusion and exclusion criteria for the review and how studies were grouped for the syntheses.	Section 2.2 (pages 4–5) – describes inclusion/exclusion criteria: exclusion of studies focused solely on synthetic polymers without bio-based component, groundwater/soil remediation, photocatalytic degradation without adsorption, and gas-phase/non-aqueous adsorption studies.
Information sources	6	Specify all databases, registers, websites, organisations, reference lists and other sources searched or consulted to identify studies. Specify the date when each source was last searched or consulted.	Section 2.1 (page 3) – Scopus database was searched on July 1, 2026. Covers period 2010–2026, with a broader search from 1974–2026 for historical context.
Search strategy	7	Present the full search strategies for all databases, registers and websites, including any filters and limits used.	Section 2.1 (page 3) – presents the complete Boolean search string with five conceptual dimensions: (1) functionalization, (2) bio-based materials, (3) polymer matrices, (4) aqueous systems, and (5) remediation processes.
Selection process	8	Specify the methods used to decide whether a study met the inclusion criteria of the review, including how many reviewers screened each record and each report retrieved, whether they worked independently, and if applicable, details of automation tools used in the process.	Section 2.2 (page 4) – describes the systematic multi-stage screening approach, including removal of duplicates, automation-flagged records, and exclusions based on predefined criteria. Figure 1 provides the PRISMA flow diagram.
Data collection	9	Specify the methods used to collect data from reports, including how many reviewers collected	Not explicitly detailed in the manuscript. A statement such as "Data extraction was

Section and Topic	Item #	Checklist item	Location where item is reported
process		data from each report, whether they worked independently, any processes for obtaining or confirming data from study investigators, and if applicable, details of automation tools used in the process.	performed independently by two reviewers, with disagreements resolved by consensus" could be added to Section 2.2.
Data items	10a	List and define all outcomes for which data were sought. Specify whether all results that were compatible with each outcome domain in each study were sought (e.g. for all measures, time points, analyses), and if not, the methods used to decide which results to collect.	Sections 2.3–2.4 (pages 5–6) – describes data items: annual publication counts, citation metrics, author productivity, keyword co-occurrence, adsorption capacities (qmax), kinetic/isotherm parameters, mechanical properties, reusability, and environmental/economic indicators.
	10b	List and define all other variables for which data were sought (e.g. participant and intervention characteristics, funding sources). Describe any assumptions made about any missing or unclear information.	Not explicitly detailed. Assumptions about missing data are not stated; this could be added as a brief statement in Section 2.2 or 2.4.
Study risk of bias assessment	11	Specify the methods used to assess risk of bias in the included studies, including details of the tool(s) used, how many reviewers assessed each study and whether they worked independently, and if applicable, details of automation tools used in the process.	Not applicable – as a bibliometric/systematic review, this item is not addressed. A statement such as "Risk of bias assessment was not performed as this review focuses on bibliometric trends and performance synthesis, not on individual study effect estimates" could be added if required.
Effect measures	12	Specify for each outcome the effect measure(s) (e.g. risk ratio, mean difference) used in the synthesis or presentation of results.	Not applicable (no meta-analysis). However, the manuscript reports descriptive and quantitative metrics: adsorption capacities (mg/g), removal efficiencies (%), growth rate (R_0 = 96.5%), R^2, F-values, tensile strength, permeability, etc.
Synthesis methods	13a	Describe the processes used to decide which studies were eligible for each synthesis (e.g. tabulating the study intervention characteristics and comparing against the planned groups for each synthesis (item #5)).	Section 2.2 (pages 4–5) – describes the screening process; Section 2.4 (pages 5–6) – describes how studies were grouped for thematic analysis (e.g., cellulose, chitosan, alginate, PVDF-biomass).
	13b	Describe any methods required to prepare the data for presentation or synthesis, such as handling of missing summary statistics, or data conversions.	Not explicitly stated. Could be added to Section 2.3 or 2.4.
	13c	Describe any methods used to tabulate or visually display results of individual studies and syntheses.	Section 2.3 (page 5) – mentions use of bibliometrix, VOSviewer, and Plotly for network visualization, co-occurrence maps, and interactive graphics. Tables 1–5 and Figures 1–2 are used for tabulation and visualization.
	13d	Describe any methods used to synthesize results and provide a rationale for the choice(s). If meta-analysis was performed, describe the model(s), method(s) to identify the presence and extent of	Not applicable (no meta-analysis). Section 2.3 (page 5) describes tools used for bibliometric synthesis (R, bibliometrix, VOSviewer, Plotly).

Section and Topic	Item #	Checklist item	Location where item is reported
		statistical heterogeneity, and software package(s) used.	
	13e	Describe any methods used to explore possible causes of heterogeneity among study results (e.g. subgroup analysis, meta-regression).	Not applicable (no meta-analysis).
	13f	Describe any sensitivity analyses conducted to assess robustness of the synthesized results.	Not applicable (no meta-analysis).
Reporting bias assessment	14	Describe any methods used to assess risk of bias due to missing results in a synthesis (arising from reporting biases).	Not applicable.
Certainty assessment	15	Describe any methods used to assess certainty (or confidence) in the body of evidence for an outcome.	Not applicable.
**RESULTS**			
Study selection	16a	Describe the results of the search and selection process, from the number of records identified in the search to the number of studies included in the review, ideally using a flow diagram.	Section 2.2 (page 4) and Figure 1 (PRISMA Flow Diagram) – describes the complete selection process: 5,008 records identified, 10 duplicates removed, 16 marked ineligible, 121 outside timeframe, 39 excluded for no bio-based component, 8 not retrieved, 26 excluded for groundwater/soil, 48 for no adsorption component, 114 for gas-phase/non-aqueous, yielding 4,626 studies included.
	16b	Cite studies that might appear to meet the inclusion criteria, but which were excluded, and explain why they were excluded.	Section 2.2 (page 4) – lists categories of excluded studies (groundwater/soil remediation, photocatalytic-only, gas-phase/non-aqueous) without citing specific studies. Individual excluded studies are not cited; this is generally acceptable for a high-level systematic review.
Study characteristics	17	Cite each included study and present its characteristics.	Table 1 (pages 8–9) – cites 10 representative publications with characteristics (cited count, source, year, document type, first author). Additional studies are cited throughout Section 3 (Results and Discussion) and Tables 2–5.
Risk of bias in studies	18	Present assessments of risk of bias for each included study.	Not applicable (not performed).
Results of individual studies	19	For all outcomes, present, for each study: (a) summary statistics for each group (where appropriate) and (b) an effect estimate and its precision (e.g. confidence/credible interval), ideally using structured tables or plots.	Tables 1–5 (pages 8–13) – present summary statistics: adsorption capacities (mg/g), removal efficiencies (%), kinetic parameters, F-values, R^2 values, circular readiness (%), resource recovery (%), energy metrics, etc., for individual studies cited in the tables and text.
Results of syntheses	20a	For each synthesis, briefly summarise the characteristics and risk of bias among contributing studies.	Not applicable (no meta-analysis). However, the synthesis in Section 3 summarizes key findings from the compiled studies.

PRISMA 2020 Checklist

Section and Topic	Item #	Checklist item	Location where item is reported
	20b	Present results of all statistical syntheses conducted. If meta-analysis was done, present for each the summary estimate and its precision (e.g. confidence/credible interval) and measures of statistical heterogeneity. If comparing groups, describe the direction of the effect.	Not applicable (no meta-analysis). The bibliometric analysis presents descriptive statistics: growth rate (R_0 = 96.5%, R^2 = 91.1%), F-values, and other metrics derived from the analyzed dataset.
	20c	Present results of all investigations of possible causes of heterogeneity among study results.	Not applicable.
	20d	Present results of all sensitivity analyses conducted to assess the robustness of the synthesized results.	Not applicable.
Reporting biases	21	Present assessments of risk of bias due to missing results (arising from reporting biases) for each synthesis assessed.	Not applicable.
Certainty of evidence	22	Present assessments of certainty (or confidence) in the body of evidence for each outcome assessed.	Not applicable.
**DISCUSSION**			
Discussion	23a	Provide a general interpretation of the results in the context of other evidence.	Section 4 (Future Research Directions, pages 13–15) and Section 5 (Conclusions, pages 15–16) – interpret findings in the context of the literature, highlighting knowledge gaps, opportunities, and contributions to circular economy.
	23b	Discuss any limitations of the evidence included in the review.	Section 4 (pages 13–15) – discusses gaps such as scarce integration of biomass with PVDF, limited scale-up studies, need for comprehensive economic and environmental impact assessments, and lack of validation in complex water matrices.
	23c	Discuss any limitations of the review processes used.	Not explicitly stated. Could be added to Section 5 or as a dedicated paragraph – e.g., limitations include reliance on a single database (Scopus), exclusion of non-English or gray literature, and potential selection bias in the screening process.
	23d	Discuss implications of the results for practice, policy, and future research.	Section 4.1–4.5 (pages 13–15) – provides detailed future research directions; Section 5 (page 15) – summarizes implications for policy (circular economy, SDGs), practice (bio-based adsorbents as superior alternatives), and future research (bridging scaling and durability gaps).
**OTHER INFORMATION**			
Registration and protocol	24a	Provide registration information for the review, including register name and registration number, or state that the review was not registered.	Section 2.1 (page 3) – states: "The review protocol was registered on the Open Science Framework (OSF) prior to data extraction" and provides the link: https://osf.io/8bcrm/overview?view_only=095f1d82b3d84d08b0e342a26898814f.

PRISMA 2020 Checklist

Section and Topic	Item #	Checklist item	Location where item is reported
	24b	Indicate where the review protocol can be accessed, or state that a protocol was not prepared.	Section 2.1 (page 3) – provides the OSF link where the protocol can be accessed.
	24c	Describe and explain any amendments to information provided at registration or in the protocol.	Not stated. This could be added (e.g., "No amendments to the registered protocol were made").
Support	25	Describe sources of financial or non-financial support for the review, and the role of the funders or sponsors in the review.	Funding section (page 16) – states "Not applicable."
Competing interests	26	Declare any competing interests of review authors.	Conflicts of Interest section (page 16) – states "The authors declare no conflicts of interest."
Availability of data, code and other materials	27	Report which of the following are publicly available and where they can be found: template data collection forms; data extracted from included studies; data used for all analyses; analytic code; any other materials used in the review.	Data Availability Statement (page 16) – states "No new data were created or analyzed in this study. Data sharing is not applicable to this article." The OSF registration link may serve as the location for protocol and supplementary materials.

From: Page MJ, McKenzie JE, Bossuyt PM, Boutron I, Hoffmann TC, Mulrow CD, et al. The PRISMA 2020 statement: an updated guideline for reporting systematic reviews. BMJ 2021;372:n71. doi: 10.1136/bmj.n71. This work is licensed under CC BY 4.0. To view a copy of this license, visit https://creativecommons.org/licenses/by/4.0/
